# Effect of Silver Nanoparticles on Biofilm Formation and EPS Production of Multidrug-Resistant *Klebsiella pneumoniae*

**DOI:** 10.1155/2020/6398165

**Published:** 2020-04-19

**Authors:** Muhammad Hussnain Siddique, Bilal Aslam, Muhammad Imran, Asma Ashraf, Habibullah Nadeem, Sumreen Hayat, Mohsin Khurshid, Muhammad Afzal, Imran Riaz Malik, Mudassar Shahzad, Umber Qureshi, Zia Ul Haq Khan, Saima Muzammil

**Affiliations:** ^1^Department of Bioinformatics and Biotechnology, Government College University, Faisalabad, Pakistan; ^2^Department of Microbiology, Government College University, Faisalabad, Pakistan; ^3^Department of Environmental Sciences, COMSATS University Islamabad, Vehari-Campus, 61100 Vehari, Pakistan; ^4^Department of Zoology, Government College University, Faisalabad, Pakistan; ^5^Department of Biotechnology, University of Sargodha, University Road Sargodha, Pakistan

## Abstract

Antibiotic resistance against present antibiotics is rising at an alarming rate with need for discovery of advanced methods to treat infections caused by resistant pathogens. Silver nanoparticles are known to exhibit satisfactory antibacterial and antibiofilm activity against different pathogens. In the present study, the AgNPs were synthesized chemically and characterized by UV-Visible spectroscopy, scanning electron microscopy, and X-ray diffraction. Antibacterial activity against MDR *K. pneumoniae* strains was evaluated by agar diffusion and broth microdilution assay. Cellular protein leakage was determined by the Bradford assay. The effect of AgNPs on production on extracellular polymeric substances was evaluated. Biofilm formation was assessed by tube method qualitatively and quantitatively by the microtiter plate assay. The cytotoxic potential of AgNPs on HeLa cell lines was also determined. AgNPs exhibited an MIC of 62.5 and 125 *μ*g/ml, while their MBC is 250 and 500 *μ*g/ml. The production of extracellular polymeric substance decreased after AgNP treatment while cellular protein leakage increased due to higher rates of cellular membrane disruption by AgNPs. The percentage biofilm inhibition was evaluated to be 64% for *K. pneumoniae* strain MF953600 and 86% for MF953599 at AgNP concentration of 100 *μ*g/ml. AgNPs were evaluated to be minimally cytotoxic and safe at concentrations of 15-120 *μ*g/ml. The data evaluated by this study provided evidence of AgNPs being safe antibacterial and antibiofilm compounds against MDR *K. pneumoniae*.

## 1. Introduction

Antimicrobial resistance has been identified as one of the three major problems countered in human health by the World Health Organization (WHO). This problem has emerged globally due to indiscriminate and extensive use of antibiotics. The spread of multidrug resistance among pathogens was mainly due to mobile genetic elements, horizontal gene transfer, transformation, and transduction [1].

*Klebsiella pneumoniae* is one of the major threats to the healthcare and gaining public interest as a nosocomial pathogen showing alarming multidrug resistance worldwide. *K. pneumoniae* is also known for its biofilm forming ability having various virulence factors such as type 1 and type 3 fimbriae, lipopolysaccharides, and outer membrane proteins that might contribute to its evasion of the immune system during infection and biofilm formation [2]. It was first found to be resistant against *β* lactams due to the production of extended-spectrum beta-lactamases (ESBLs) [3, 4]. ESBLs producing *K. pneumoniae* are also found to be resistant against other antibiotics such as quinolones that can cause treatment failure. Multidrug resistance in *K. pneumoniae* became a worldwide threat for human health with high mortality rates and less treatment options. Under such situations, multidrug-resistant *K. pneumoniae* can only be treated by tigecycline and colistin that are the last resorts of antibacterial drugs [3].

Biofilms are structured aggregates of bacteria capable of surviving hostile environmental conditions and exhibit resistance to the host's immunity and different chemotherapeutic agents [5]. Biofilms are complex of single or multiple species of bacteria enclosed in an extracellular polymeric substance (EPS) that is mainly composed of polysaccharides, nucleic acids, and proteins [6, 7]. Since infections caused by biofilm-forming bacteria are difficult to treat, therefore, it is a need of this time to search for novel biofilm inhibitors.

Silver nanoparticles (AgNPs) are known to have antibacterial effects against pathogenic bacteria and also against bacteria exhibiting resistance against antibiotics. In the past few years as antibiotic resistance has emerged as a major health concern globally [8], there has been a serious demand for the discovery of alternatives for the treatment of drug-resistant microbial infections apart from antibiotics. Nanoparticles having a wide range of applications due to their smaller size and higher surface area to volume ratio are now being studied extensively for their antibacterial and antibiofilm effects [9]. AgNPs are one of the primary nanoparticles that were observed for their extraordinary potential to combat pathogenic multidrug bacterial isolates as an alternative approach to treat bacterial infections [10]. However, the role of AgNPs as efficient biofilm inhibitor and their effect on extracellular polymeric substance (EPS) production have not yet been given sufficient attention.

Therefore, the present study was designed to evaluate the antibacterial and antibiofilm efficacy of the AgNPs against clinical isolates of multidrug-resistant *Klebsiella pneumoniae* MF953599 and MF953600. The biostatic and bactericidal effects of the AgNPs against *K. pneumoniae* were conducted to determine the minimum inhibitory concentration (MIC) and the minimum bactericidal concentration (MBC). The production of extracellular polymeric substance (EPS) and antibiofilm activity was assessed in the presence of the subinhibitory concentrations of AgNPs. Finally, the cytotoxicity potential of the AgNPs was evaluated against HeLa cell lines by neutral red uptake assay.

## 2. Materials and Methods

### 2.1. Bacterial Strains and Reagents

Multidrug-resistant (MDR) clinical isolates of *Klebsiella pneumoniae* MF953599 and MF953600 [11] were procured from the Department of Microbiology, Government College University Faisalabad (GCUF), Pakistan. The bacterial cultures were maintained in Luria-Bertani (LB) broth and agar (Merck, Germany). The chemicals that are used in the present study were obtained from Sigma-Aldrich (St. Louis, MO, USA) and Merck (Darmstadt, Germany) else stated. For synthesis of AgNPs, silver nitrate (AgNO_3_) was used as a progenitor, polyvinyl pyrrolidone (PVP) as protective agent, and glucose as a reducing agent, and sodium hydroxide (NaOH) was used as a velocity accelerator of the chemical reaction.

### 2.2. Synthesis of AgNPs

The silver nanoparticles (AgNPs) were synthesized by wet method. 0.1 M solution of AgNO_3_ (50 ml) was prepared in distilled water, and the contents were mixed thoroughly by a magnetic stirrer. Another 0.1 M solution (50 ml) of sodium borohydride (NaBH_4_) was prepared, and polyvinyl pyrrolidone (PVP) was used for stabilization of silver nanoparticles. 50 ml of AgNO_3_ solution was titrated with 50 ml of NaBH_4_ solution dropwise. The mixture was constantly stirred and centrifuged at 6000 rpm for 10 minutes to separate the AgNPs. The AgNPs were oven dried at 100°C for 36 h and stored for further application.

### 2.3. Characterization of AgNPs

The AgNPs were characterized through UV-Visible spectroscopy, X-ray diffraction (XRD), and scanning electron microscopy (SEM) as described by Zhang et al. [12]. For UV-Vis spectroscopy, AgNP concentration (5 mg/20 ml) was prepared by diluting nanoparticles in deionized water and their absorbance was measured in a wavelength range 300-700 nm to find the wavelength for maximum absorbance. The crystalline size of the AgNPs was measured with an analytical X'Pert, X-ray diffractometer using CuK*α*1 radiations (*λ* = 1.540598 Å), at 40 kV and 30 mA. The 2*θ* range was acquired from 30° to 80°, and JCPDS Cards were used as standards to find the respective phases of the particles. The crystallite size was calculated by using the Debye-Scherrer equation. For SEM images, dried particles were mounted on an aluminum stub and coated with gold to get better contrast. SEM analysis was performed with a scanning electron microscope (JEOL JSM-6480).

### 2.4. Antibacterial Susceptibility Assay of AgNPs

The susceptibility of multidrug-resistant *K. pneumoniae* against AgNPs was determined by agar diffusion method as described by [13]. Bacterial isolates were cultured in LB broth overnight, and turbidity was adjusted to 5 × 10^5^ CFU/ml. The bacterial inoculum was plated on LB agar medium (Sigma-Aldrich, St. Louis, MO, USA). Wells having a diameter of 5 mm were made aseptically on plates, and 0.1 ml of various concentrations of AgNPs (1000 *μg*/*ml*, 500 *μg*/*ml*, 250 *μg*/*ml*, and 100 *μg*/*ml*) was dispensed in wells with the help of micropipette followed by overnight incubation at 37°C. After incubation, the diameters of zones of inhibition were measured. The wells containing dimethyl sulfoxide (DMSO, 0.1%) alone served as a negative control.

### 2.5. Evaluation of Bacteriostatic Potential of AgNPs

The bacteriostatic potential of AgNPs was evaluated using broth microdilution method. Twofold serial dilutions of AgNPs (0-1000 *μ*g/ml) in Mueller-Hinton broth were made using a 96-well microtiter plate (Thermo Fisher Scientific, Rockford, IL, USA). 18-24 hours bacterial inoculum with turbidity adjusted according to 5 × 10^5^ CFU/ml was dispensed in wells containing different concentrations of AgNPs and incubated at 37°C for 24 hours. Following incubation, bacterial cell viability was assessed by a change in color from yellow to blue after the addition of redox dye nitro-blue tetrazolium chloride (NBT). The lowest concentration that did not change the color of the dye was considered as MIC against the target bacteria. The experiment was performed in triplicate and negative control was also included.

### 2.6. Evaluation of Bactericidal Potential of AgNPs

Minimum bactericidal concentration (MBC) was determined by preparing bacterial inoculum with turbidity adjusted to 5 × 10^5^ CFU/ml, and it was further added in LB broth having AgNP concentrations equal to MIC and above. The broth was incubated for 24 hours at 37°C, and 100 *μ*l of the broth from each dilution was spread over the surface of LB agar plate. After 24 hours of incubation at 37°C, the plates were checked for the appearance of visible growth. The lowest concentration of AgNPs that gave no visible growth on agar plates was termed as MBC.

### 2.7. Determination of Cellular Protein Leakage

The cellular protein leaked due to the bacterial death and disruption of the cell envelope was estimated by the Bradford assay (Bradford, 1976). Cells grown to a final density of 5 × 10^5^ CFU/ml were inoculated into LB media containing various concentrations of AgNPs (0.5 × MIC, 1 × MIC, 1.5 × MIC) for both of the bacterial strains, while no AgNPs were added in control. After incubation at 37°C for 12 hours, 1 ml from all the cultures was subjected to the centrifugation for 30 minutes at 300g for harvesting the supernatant that was further used for measuring the quantity of cellular protein through the Bradford assay by measuring the absorbance at 595 nm.

### 2.8. Fourier Transform Infrared Spectroscopy (FTIR)

FTIR spectra of *Klebsiella pneumoniae* strain MF953599 with and without AgNPs were obtained in transmission mode in wavenumber range 500-4000 cm^−1^. Strain MF953599 was cultured for 24 hours and then cells were harvested by centrifugation at 6000 × g for 15 minutes, and after washing with 1x PBS (phosphate buffer saline), pellets were placed in bench top freeze drier (Labconco™, Missouri, United States of America) overnight. The dried cell pellets were used for infrared spectroscopic analysis and spectra were recorded on FTIR spectrometer (Bruker, Massachusetts, United States of America). The FTIR spectrum was recorded in the region 4000 to 500 cm^−1^ wave number.

### 2.9. Effect of AgNPs on the Production of Extracellular Polymeric Substance (EPS)

In order to investigate the effect of AgNPs on the production of extracellular polymeric substances (EPS), overnight bacterial suspension in LB broth was prepared and was further diluted to adjust its turbidity according to 0.5 McFarland Standards to achieve final concentration of 5 × 10^5^ CFU/ml. 2 ml of the inoculum was added in 100 ml of LB broth having AgNPs at subinhibitory concentrations. Control was prepared without the addition of AgNPs. Both flasks were incubated at 37°C for 24 hours on a shaking incubator. After incubation, EPS was extracted. For that purpose, bacterial culture was centrifuged at 6000 rpm for 30 minutes at 4°C and the supernatant was collected. Two volumes of acetone were added to the supernatant, and the mixture was refrigerated overnight (at 4°C) for the precipitation of EPS. EPS product was finally collected by centrifugation of the mixture at 6000 rpm for 30 minutes (at 4°C) to collect pellet. Wet weight of the pellet was measured and dry weight was estimated after drying it at 40°C for 24 hours [14].

### 2.10. Biofilm Formation Assay

The antibiofilm activity of the AgNPs was determined qualitatively by tube method [15]. 50 *μ*l of the overnight culture of the targeted bacteria in LB broth was further diluted to adjust its turbidity according to 0.5 McFarland Standards (5 × 10^5^ CFU/ml). The suspension was added in the tubes containing 2 ml of sterilized LB broth, and these tubes were incubated at 37°C for 24 hours after the addition of various concentrations of AgNPs (25, 50, 75, and 100 *μ*g/ml) in separate tubes. Negative control without the presence of the bacterial suspension and the positive control that was left without the addition of AgNPs were also included in the experiment. After incubation, the broth culture was decanted and washed twice with PBS. The inside of the tubes was stained with crystal violet dye (0.1%) for half an hour, while the excess dye was decanted and washed off with deionized water gently. The tubes were dried and the biofilm formation ability was determined by observing a thin layer of blue film on the walls of tube.

For quantitative estimation of biofilm formation, the microtiter plate assay was used [15]. In this method, 96-well microtiter plates were used and wells were inoculated with 180 *μ*l of LB broth, 10 *μ*l of culture grown overnight and further diluted to adjust its final concentration to 5 × 10^5^ CFU/ml, and 10 *μ*l of AgNPs (concentrations used were 0-100 *μ*g/ml). After incubation at 37°C for 24 hours, the content of the wells was removed gently and washed thrice with PBS to remove bacterial cells. After that, biofilms were fixed with sodium acetate followed by staining with crystal violet dye (0.1%) for 10 minutes. The stained cells attached to wells were then washed with distilled water and dried. 200 *μ*l of 95% ethanol was added in each well to elute the attached cells, and absorbance was measured at 620 nm on ELISA reader in order to quantify cells capable of forming biofilms. Negative and positive controls were also used in the assay using sterile growth medium only and working solution, respectively.

The %age of biofilm inhibition was calculated by the following formula:

(1) $$\begin{array}{c} \%\text{age biofilm inhibition}=1-\frac{\text{OD}620\text{ of cells treated with AgNPs}}{\text{OD}620\text{ of nontreated control}}\times 100. \end{array}$$

### 2.11. Cytotoxic Potential of AgNPs

The cytotoxic potential of AgNPs was estimated by neutral red uptake assay using HeLa cell lines following the method described by [16]. Cells were grown in Dulbecco's modified Eagle's high-glucose medium (Gibco, Gaithersburg, MD, USA) provided with 1% antibiotics (penicillin/streptomycin), 10% FBS, and incubated for 24 hours in the presence of CO_2_ (5%) and humidity (90%). After incubation, the medium was changed and the cells were again incubated for 24 hours in the presence of different concentrations of AgNPs (0-250 *μ*g/ml) along with control (without NPs). Media were removed after incubation, and the cells were washed with PBS (phosphate buffer saline). Then, 100 *μ*l of neutral red media (40 *μ*g/ml) was added in each well and the cells were incubated again at 37°C for 2 hours. After incubation, neutral red media were removed and the cells were washed again with PBS followed by destaining with 150 *μ*l of freshly prepared destaining solution (49% water, 50% absolute ethanol, and 1% glacial acetic acid). The microtiter plate was shaken on a microtiter plate shaker for a period of 10 minutes. The absorbance was then measured at 540 nm using a microtiter plate reader. In order to get the digital images of the control and treated cells, phase-contrast microscopy of the HeLa cells was also performed using an attached Charge-Coupled Device (CCD) SP 480 H color camera (Olympus, Japan).

### 2.12. Statistical Analysis

All the experiments were performed in triplicates and the results were expressed as means ± SE. In order to check the significance of each experiment, Student's *t*-test and ANOVA (analysis of variance) were performed using Microsoft Excel. A value of *p* < 0.05 was considered to be statistically significant.

## 3. Results

**Figure 1:**
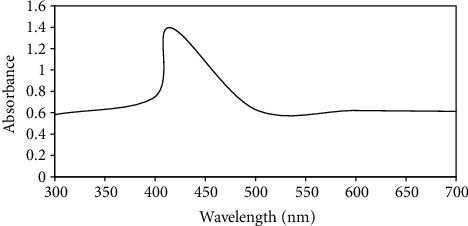
UV-Visible absorption spectrum of AgNPs showing absorbance peak at the wavelength of 415 nm.

The results of UV-Visible spectroscopy (Figure 1) revealed that the synthesized AgNPs exhibited a well-defined plasmon band at the wavelength of 415 nm with an absorbance value of 1.397. The symmetrical shape of the plasmon band indicated the sharp particle size distribution with smaller particle size.

**Figure 2:**
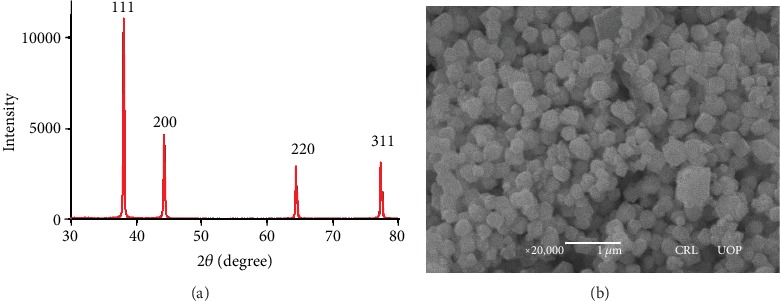
XRD spectra of AgNPs synthesized by chemical reduction method (a); SEM micrograph of AgNPs (b).

In XRD analysis, the peak corresponding to (111) is more intense than other planes. The crystalline size of AgNPs obtained from the X-ray diffraction spectrum given in Figure 2(a) was 20 nm, while SEM micrograph of AgNPs indicated that the prepared NPs were more or less spherical in shape with an average diameter of around 50-65 nm as shown in Figure 2(b).

The effect of various concentrations of AgNPs on *K. pneumoniae* was determined by agar well diffusion assay. The results revealed that NPs showed antibacterial activities in a dose-dependent manner since the diameter of zone of inhibition was increased by increasing the concentrations of AgNPs. As demonstrated in the results, the diameter of zones of inhibition was in the range of 12-37 mm. Maximum inhibition in terms of the dimeter of zone of inhibition was observed against *K. pneumoniae* MF953599 and MF953600 (Table 1).

**Figure 3:**
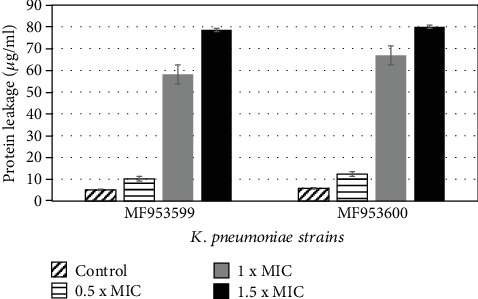
Effect of silver nanoparticles on membrane protein leakage. The Bradford method was used to detect the effect of different concentrations of NPs on leakage of protein. Values are presented as mean + SE.

To evaluate the susceptibility of *Klebsiella pneumoniae* towards AgNPs, MIC and MBC values were determined. For the evaluation of MICs, broth microdilution assay was used. The results indicated that the MIC value of NPs against *Klebsiella pneumoniae* MF953599 was 125 *μ*g/ml while for *K. pneumoniae* MF953600 the MIC value was found to be 62.5 *μ*g/ml. The data revealed that the MBC value for the bacteria used in the current study was in the range of 250-500 *μ*g/ml (Table 2). In order to investigate the effect of AgNPs on the leakage of cytoplasmic proteins, the content of protein released into the supernatant was quantified by the Bradford method. The results have indicated that cells treated with AgNPs (at 1 × MIC and 1.5 × MIC) showed a significant (*p* < 0.001) increase in the protein leakage as compared to the control and cells treated with subinhibitory concentrations (0.5 × MIC) of AgNPs (Figure 3). The data revealed that after 12 hours of incubation in the presence of NPs the amount of protein released into the supernatant was increased thus showing an increase in the membrane disruption (Figure 3).

**Figure 4:**
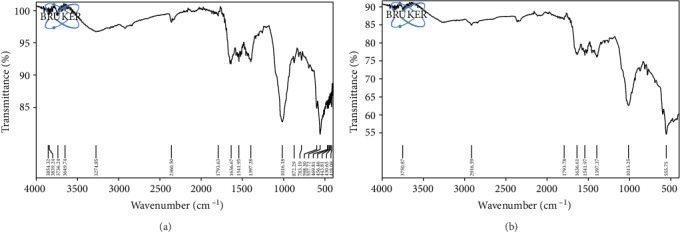
FTIR spectrum of *Klebsiella pneumoniae* strain MF953600: (a) control cells and (b) with AgNP treatment.

Infrared spectrum of *K. pneumoniae* revealed the presence of carbonyl (>C=O), carboxyl (-COOH), amide (-NH_2_), hydroxyl (-O-H), and alkyl (-C-H) groups in its cell envelop. The region between 710 and 1490 cm^−1^ indicated the presence of carbohydrate moieties specifically complex polysaccharides. The absorption bands at 783.19 cm^−1^, 872.29 cm^−1^, and 1016.18 cm^−1^ showed the existence of polysaccharide sugars like glucans and mannans. The spectrum revealed the strong absorption of the amide group (1541.95 cm^−1^), asymmetric stretching of the C=O group (1636.67 cm^−1^), weak stretching of the C-H group (2360.50 cm^−1^ and 2916.59 cm^−1^), and strong stretching of the O-H group (3274.85 cm^−1^) as shown in Figure 4. According to our findings, silver nanoparticles interacted with the cell envelope by hydrogen bonding, which resulted in the shifting of spectral band positions at 557.72 cm^−1^ ⟶ 555.75 cm^−1^, 783.19 cm^−1^ ⟶ 782.30 cm^−1^, 1016.18 cm^−1^ ⟶ 1011.33 cm^−1^, 1636.67 cm^−1^ ⟶ 1636.09 cm^−1^, and 3274.85 cm^−1^ ⟶ 3273.40 cm^−1^ (Figures 4(a) and 4(b)).

**Figure 5:**
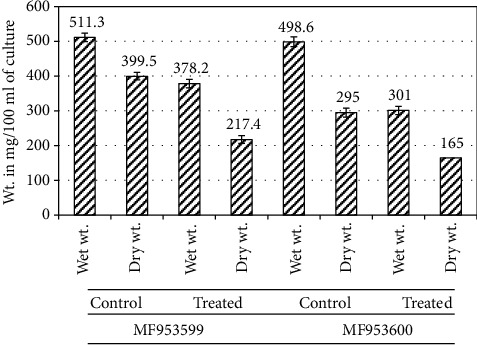
Quantification of EPS extracted from bacterial cells in the presence and absence of subinhibitory concentrations of AgNPs. The results are expressed in terms of wet and dry weights of EPS extracted.

To determine the effect of AgNPs on the production of extracellular polymeric substances (EPS), *Klebsiella pneumoniae* strains were grown in the presence and absence of subinhibitory concentrations of AgNPs. The results for the wet and dry weights of the extracted EPS indicated that as compared to the control cells there was a significant (*p* < 0.05) decrease in the amount of EPS from the cultures treated with NPs. As indicated in Figure 5, there was a 26% decrease in the wet weight of EPS and nearly 45.5% decrease in the dry weight of EPS extracted from *K. pneumoniae* strain MF953599 after treatment with subinhibitory concentrations of AgNPs, while for strain MF953600, nearly 40% decrease in the wet weight of EPS and around 44.05% decrease in the dry weight of extracted EPS were observed in the presence of NPs as compared to control cells (Figure 5).

**Figure 6:**
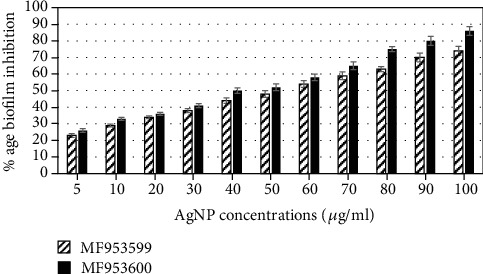
Percent inhibition of biofilm formation by various concentrations of AgNPs against *Klebsiella pneumoniae*. For quantification of biofilm formation, absorbance was measured at 620 nm.

The effect of AgNPs directly on the formation of biofilms was estimated by tube method by culturing *Klebsiella pneumoniae* in the presence of different concentrations of AgNPs. The results were indicated by the development of thin layer of biofilms after staining with dye. In the case of *K. pneumoniae* strain MF953599, it was observed that no visible biofilm formation was seen in culture tubes having AgNPs at a concentration of 100 *μ*g/ml, while in the case of strain MF953600, no visible biofilm formation was revealed at the concentrations of 75 *μ*g/ml or above (Table 3). The quantitative estimation of biofilm formation was done by the microtiter plate assay, and % inhibition of biofilms in *Klebsiella pneumoniae* after treatment with different concentrations of NPs was also investigated. The results indicated that in most of the cases treatment of cells with subinhibitory concentrations of AgNPs significantly (*p* < 0.05) reduced biofilm formation in a dose-dependent manner. As shown in Figure 6, % inhibition of biofilm formation was 74% for strain MF953599 at the highest concentration tested (100 *μ*g/ml), while for strain MF953600, the inhibition was found to be 86%.

**Figure 7:**
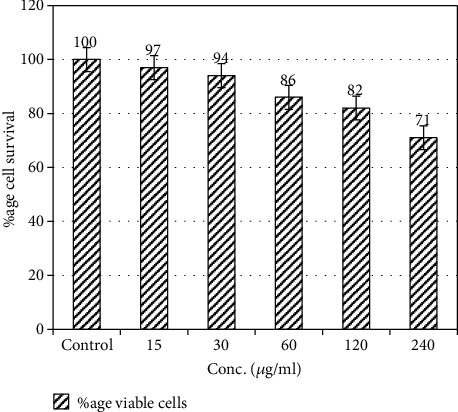
% viability of HeLa cells treated with different concentrations of AgNPs. 100% value represents zero toxicity whereas lower values are depicting cytotoxicity towards HeLa cell lines. Error bars represent standard error from the mean of triplicate experiments.

The cytotoxic effect of AgNPs on mammalian cell lines was determined by neutral red uptake assay after incubating HeLa cell lines. The results were determined as % viability of cells in the presence of different concentrations of AgNPs. It was observed that there was no significant effect (*p* > 0.05) in terms of % viability of HeLa cell lines up to the concentrations of 120 *μ*g/ml, since the % viability of the cells was found to be 82-97% as compared to untreated or control cells (where 100% cell viability was observed). At the highest concentration tested, % viability was found to be 71% (as shown in Figure 7).

**Figure 8:**
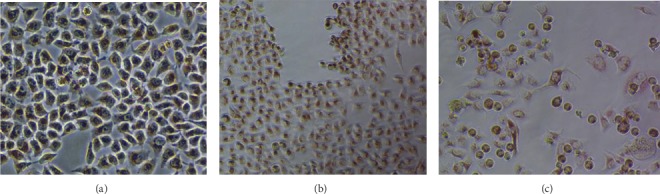
Phase-contrast microscopy of HeLa cells treated with different concentrations of silver nanoparticles. (a) Untreated cells (control). (b) Cells treated with NPs at a concentration of 60 *μ*g/ml. (c) Cells treated with NPs at a concentration of 240 *μ*g/ml showing morphological deformities such as lysis and rounding off.

Phase-contrast microscopy indicated that AgNPs were nontoxic to HeLa cells at various concentrations tested (15-60 *μ*g), while at the highest concentrations (240 *μ*g/ml), the cytotoxic effects in terms of morphological deformities such as cell rounding and lysis were observed (Figure 8).

## 4. Discussion

The present study suggests that AgNPs can be utilized as efficient therapeutic and antibiofilm agents against MDR *Klebsiella pneumoniae*. We synthesized spherical-shaped AgNPs by chemical reduction method having a diameter of around 50-65 nm. In another study by Suriati et al. [17], AgNPs were produced with a diameter ranging from 35 to 80 nm by using trisodium citrate as a reducing agent and ascorbic acid as a surfactant [18]. Nanoparticles can be efficiently used as antibacterial agents against numerous pathogens. Our data showed that AgNPs have antibacterial potential against multidrug-resistant *K. pneumoniae* strains as suggested by MIC and MBC values (Table 2). Previous studies also suggested that these NPs could be used as promising antibacterial agents against a number of bacterial pathogens including *K. pneumoniae* [19]. The exact mechanism of antibacterial action of AgNPs is still unclear; however, various mechanisms have been proposed. One of the suggested mechanisms is the ability of these NPs to anchor cell wall of bacteria and their subsequent penetration inside the cell, thus inducing structural changes in the cellular membrane that is responsible for the leakage of cellular content and cell lysis [20]. The present study clearly indicated that AgNPs have significant potential to disrupt cell membrane thus causing leakage of content that was detected by the quantification of membrane proteins leaked out from the cells (Figure 3). FTIR spectrum of the tested strain also suggested loss of numerous functional groups (present in polysaccharides and protein) that were present in the cellular envelope (Figure 4).

Biofilm-forming microbes have the ability to cause various diseases, and according to one of the reports presented by the National Institutes of Health and Centre of Disease Control, biofilm-forming microbes are responsible for causing 65-80% infections [21]. Thus, one of the efficient strategies to control the infections by these microbes is to use biofilm inhibitors. Various studies have indicated the effective role of NPs as biofilm inhibitors against target bacteria. One of our previous studies indicated the antibiofilm and antiadhesion potentials of magnesium oxide nanoparticles against drug-resistant bacteria [22]. In the current study, we described the potential of AgNPs to target biofilm formation and extracellular polymeric substance (EPS) production against *Klebsiella pneumoniae*. As shown by the results, *Klebsiella pneumoniae* strain MF953600 was incapable of producing biofilm formation at the concentration of 75 *μ*g/ml (Table 3). In the present study, % inhibition of biofilm formation was also detected by the microtiter plate assay and the results indicated that there was 23-86% inhibition of biofilm formation in *Klebsiella pneumoniae* in the presence of various concentrations of AgNPs (as shown in Figure 6). Our results are consistent with the previous reports by Goswami et al. [23] who studied the effect of AgNPs on biofilm production of different bacteria and found that AgNPs are capable of 89% inhibition of biofilms formed by *S. aureus* and 75% for *E. coli* at a concentration of 15 mg/ml. Fattah et al. [24] also demonstrated the efficacy of nanosized silver (at the size of 100 nM) against *Pseudomonas aeruginosa*. Their study indicated that there was 95% reduction in the biofilm production by *Pseudomonas aeruginosa* due to disruption of EPS matrix. Our study also suggested that biofilm inhibition in *Klebsiella pneumoniae* might be because of the disruption of the EPS matrix. However, NPs can also increase the biofilm biomass in microbes. Contrary to our results, one of the previous studies by Haney et al. [25] demonstrated an increase in biofilm biomass of *Pseudomonas aeruginosa* after treatment of cells with superparamagnetic iron oxide nanoparticles at a concentration of 0.2 mg/ml. Their study suggested that iron nanoparticles could be used as a source of elemental iron by the cell that is why they observed an increase in biofilm biomass with corresponding increase in cell density.

NPs have numerous attractive features in comparison to their bulk counterparts; however, many reservations in terms of their safety towards human health have been pointed out. The cytotoxic potential of numerous antibacterial agents had hindered their establishment as promising chemotherapeutic agents. Therefore, AgNPs at various concentrations were tested for their toxic potential against HeLa cells lines using neutral red uptake assay. Our results suggested that these NPs were nontoxic up to the concentrations of 120 *μ*g/ml as shown in Figure 7; however, cytotoxic effects were observed at the highest concentration tested (240 *μ*g/ml). Our results are consistent with the findings of Kaba and Egorova [26], who also observed the nontoxic nature of silver nanoparticles.

In short, the current study clearly described the role of AgNPs as efficient antibacterial and antibiofilm agents against multidrug-resistant *Klebsiella pneumoniae*. In the future, there is a need to work on other bacterial pathogens for the eradication of their biofilms.

## 5. Conclusion

This present work emphasized the synthesis, characterization, and antibacterial properties of AgNPs. Synthesized nanoparticles were characterized by morphology properties, UV–Vis, and XRD that revealed the crystalline nature of the nanoparticles. SEM images showed the spherical shape of the AgNPs with an average size of 55 nm. Furthermore, these AgNPs were assessed for antimicrobial activity against MDR *K. pneumoniae*. AgNPs exhibited excellent antibacterial activity at 125 *μ*g/ml for MF953599 while for MF953600 at 62.5 *μ*g/ml. AgNPs exhibited antibiofilm activity against two MDR strains MF953599 and MF953600. Therefore, the nanoparticles might be useful for various fields like pharmaceutical products, water purification, drug delivery, and many other commercial processes.

## Figures and Tables

**Table 1:** Antibacterial effect of AgNPs determined by agar well diffusion method.

Strain	Diameter of zone of inhibition (mm)			
	1000 (*μ*g/ml)	500 (*μ*g/ml)	250 (*μ*g/ml)	100 (*μ*g/ml)
MF953599	34 ± 1	26 ± 2	18 ± 0.5	12 ± 1
MF953600	37 ± 0.5	27 ± 1	19 ± 1	15 ± 0.5

**Table 2:** MIC and MBC for AgNPs against *Klebsiella pneumoniae*.

Strains	MIC (*μ*g/ml)	MBC (*μ*g/ml)
*K. pneumoniae* MF953599	125	500
*K. pneumoniae* MF953600	62.5	250

**Table 3:** Qualitative estimation of biofilm formation by tube method.

Intensity of color of biofilm	Positive control	Concentrations of Ag NPs (*μ*g/ml)				Negative control
		25	50	75	100	
MF953599	++++	+++	+	+	−	−
MF953600	++++	+++	+	−	−	−

## Data Availability

The data used to support the findings of this study are included within the article.
